# Hospital Doctors under Surveillance

**Published:** 1889-10-19

**Authors:** 


### The Hospital

A Weekly Institutional Journal of

**Science, Medicine, Nursing, and Philanthropy.**

*For Hospitals, Asylums, Medical Practitioners, Students, Nurses, Families, Parishes, Congregations, and the Charitable Public.*

Vol. VII. No. 160.] [October 19, 1889.

### Hospital Doctors Under Surveillance.

The crisis which has arisen in the affairs of the City-road Chest Hospital is of a nature to excite the deepest interest in every friend and supporter of voluntary hospitals throughout the world. The question at issue involves not only the professional liberty of the honorary officers of medical charities, but the safety of the patients committed to their charge; and not only the safety of the patients, but the progress and improvement of medical science itself. But it may be thought by some that medical and surgical practice are now at such an advanced stage of efficiency that little more remains to be learnt; and that new methods must therefore be more or less of the nature of curious and hardly justifiable experiments. Nothing could be further from the truth. That medical science within the last two hundred years has advanced by leaps and bounds everyone will admit. But why has it advanced with such telegraphic rapidity? Because before that time a true science of physiology and pathology, or of healthy and diseased activity, was hardly known, and therefore, since almost the whole region of medicine was practically untravelled, it was possible to cover large tracts of new country in a very brief space of time, and thus to show progress which was little less than wonderful. It does not, however, follow that because very much has been done, nothing remains to be done. Hospitals are pre-eminently progressive medical institutions; and if any man is sure of receiving the newest, most scientific, and most effective treatment known to medical science, it is the man who has the good fortune to be treated in a hospital.

Now let these obvious and reasonable principles be applied to the facts of the present crisis at the City-road Chest Hospital. The secretary of that institution charges upon the medical staff that they take in emergency cases without hope of their living to leave the hospital, cases which are chiefly interesting from a medical and surgical point of view, and that they try experiments upon their patients. There is no evidence whatever to substantiate these accusations; but even if there were evidence to prove them up to the hilt—what then? Surely it is scarcely less the function of a hospital to alleviate the sufferings of the dying than to treat those who have a prospect of recovery. But the figures of the cases admitted do not bear out Mr. Austin's statement, for the mortality of the emergency cases has been less than that of the others. And, again, the secretary errs in charging the doctors with the selection of these cases, since the selection is made by the governors who give the letters of recommendation. Then the accusation that the doctors select cases on account of their medical and surgical interest: what does that mean? It means nothing more than this, that cases are selected because of their unusual difficulty; because, being difficult and uncommon, they are likely to fare badly at the hands of outside practitioners, whose experience lies chiefly among ordinary cases, and who seldom or never have time to devote to patients whose diseases are of a complicated or unfamiliar character. This is perfectly well known. Even the outside public require no evidence beyond their own experience to make them fully assured of it. But, as we have said, although it is perfectly natural and right that difficult and uncommon cases should be sent to hospitals, there is no evidence whatever that the City-road Hospital has ever made a single effort to bring within its walls more than the average proportion of such cases that is found at all hospitals.

With regard to the second charge, that the physicians and surgeons of the hospital are too much given to experimentation: where is the proof of it? The experimentation has, it is said, been going on for at least three years. How many cases has the accuser brought forward in support of his charge during that prolonged period? Two! And what are they? The one is a case of tracheotomy. But is tracheotomy now an experimental operation? As well might it be said that the administration of chloroform is an experimental operation, or the removal of the stumps of teeth. Tracheotomy at the present time is one of the most common, as it is one of the most successful of all major operations. There is hardly a middle-aged general practitioner in any large town in England who has not either seen elsewhere, or had in his own practice, one or more cases of tracheotomy. What is more, the vast majority of these cases when taken in time prove splendidly successful. So little is tracheotomy an "experiment" that nobody but a tyro would dream of calling it one. It may be taken for granted, absolutely, that in no well-conducted hospital would tracheotomy ever be thought of except when it is necessary for the saving of the patient's life. As an instance of the absolute recklessness of the secretary's statement may be taken the fact that he says of the house physician that he performed this operation for the first time—such statement being, we are informed, absolutely untrue.

The other operation charged against the staff was one for aneurism. Now aneurism is always difficult to treat, and the treatment is often unsuccessful. From the time of John Hunter downwards surgeons have devoted their best and most earnest thought to this dangerous casualty. The inevitable result has been that a great variety of methods of treating the mischief caused by the accident, if it can properly be called an accident, have been tried. But why have so many different methods been employed? For the simplest of all reasons, that no one method has proved itself so generally successful as to establish its claim to universal use. Certain forms of aneurism constitute almost one of the *opprobria medicinæ*. Even when aneurisms can be easily reached the treatment must always be entered upon with doubt and fear. But when they occupy internal situations, as was the case with the particular aneurism treated at the Chest Hospital, then indeed the treatment belongs almost entirely to the "kill or cure" class of operations. One thing is certain, that in the case of an aneurism so situated as the one in question, death is inevitable unless an operation be promptly performed.

Directly the honorary medical officers heard of the secretary's statements they at once showed their sense of the gravity of the situation by demanding an immediate investigation, and to leave the council free to protect the best interests of the hospital and its patients, they unreservedly placed their resignations in its hands. This action, so different from that of their assailant, coupled with the fact that after full enquiry the council unanimously passed a vote of confidence in the medical staff, and requested them to withdraw their resignations, will make it easy for all impartial and thoughtful persons to see at once who was really at fault throughout. But, strange to say, at the recent meeting of governors, although most of the speakers admitted the indiscretion of the secretary in making such statements, and eulogistically praised the members of the medical staff, the majority proceeded to vote what practically amounted to the dismissal of the staff and the retention of the secretary in office. This action goes to show that the majority of those present attached more importance to their championship of one individual, the secretary, than they did to the best interests of the institution and its patients. Although the secretary stated he made no charges, his insinuations, if they had any meaning, implied that the doctors, as Dr. Hensley said, had abused their position by causing fit applicants for relief to be excluded from the benefits of the hospital. Further, that the medical officers by admitting patients for experimental purposes, and not with the hope of doing all for them that lay in their power, were unworthy to hold office. Charges of such gravity demanded instant investigation, but, strange to say, no enquiry of any kind was asked for by the secretary. Indeed, he only made these insinuations at the end of a letter written in his own defence in reply to some grave charges against himself, not referred to at the meeting, which after a deliberate enquiry by a special committee led to his being severely censured by the council. To claim privilege for insinuations so made was to let judgment go by default, besides, being, as the council declared, unworthy and indefensible. Fortunately this case is unique in the history of British hospitals, and secretaries, as a rule, are men of too much education and good sense to so far forget themselves as to attempt a surveillance and criticism of the medical and surgical officers attached to the hospitals under their management.

